# Microbial Consortia Involved in Traditional Sicilian Sourdough: Characterization of Lactic Acid Bacteria and Yeast Populations

**DOI:** 10.3390/microorganisms10020283

**Published:** 2022-01-26

**Authors:** Alessandra Pino, Nunziatina Russo, Lisa Solieri, Laura Sola, Cinzia Caggia, Cinzia Lucia Randazzo

**Affiliations:** 1 Department of Agricultural, Food and Environment, University of Catania, 95123 Catania, Italy; alessandra.pino@unict.it (A.P.); nunziatinarusso83@gmail.com (N.R.); cinzia.caggia@unict.it (C.C.); 2 ProBioEtna srl, Spin-off of University of Catania, 95123 Catania, Italy; 3 Department of Life Sciences, University of Modena and Reggio Emilia, 42122 Reggio Emilia, Italy; lisa.solieri@unimore.it (L.S.); 203738@studenti.unimore.it (L.S.)

**Keywords:** ancient grains, microbial community, ARDRA, PCR-RFLP analysis, rep-PCR analysis

## Abstract

Sourdough is one of the oldest starters traditionally used for making baked goods, offering several advantages to the sensory, rheology, and shelf life of final products. The present study investigated, for the first time, the microbiota of spontaneously fermented Maiorca dough samples collected from bakeries located in Sicily (Italy). Four sourdough samples (M1, M2, M3, and M4), were produced using *Triticum vulgare* Host. var. *albidum* Koern (Maiorca grain) were subjected to LAB and yeasts isolation and identification at the species level. The in-depth characterization of the lactobacilli population revealed that *Lactiplantibacillus plantarum* and *Levilactobacillus brevis* unquestionably dominated the Maiorca sourdough ecosystem. Concerning the yeasts community, high species diversity was found. *Saccharomyces cerevisiae* and *Wickerhamomyces anomalus* were the most frequently isolated species. In addition, *Torulaspora delbrueckii, Pichia kluyveri, Candida boidinii,* and *Candida diddensiae* were also detected. Investigations on both pro-technological and functional traits of the isolated strains could lead to the selection of starters for the production of baked goods.

## 1. Introduction

In the last decade, the new trend towards the production and consumption of natural, traditional, and healthy foods helped to drive changes in the formulation of cereal-based products [1]. In fact, both consumers and the industry experienced increasing interest in the formulation of bakery foods using traditional sourdough [2]. 

Sourdough has been used since ancient times as a biotechnological strategy for the fermentation of cereals. Based on its microbiota, sourdough harbors a very complex microbial consortium [3], mainly resulting from the type of flour and ingredients used [4,5,6,7,8]. In addition, the applied technological parameters consistently affect the sourdough microbiota, which in turn influence the sensory, nutritional, texture, and shelf-life features of baked goods [9]. Notably, flour represents the main source of autochthonous lactic acid bacteria (LAB) in spontaneous sourdough fermentation, whose role is essential to establish a balanced microbial consortium between LAB and yeasts [10,11]. Usually, this microbial consortium consists of yeasts and obligate or facultative heterofermentative lactobacilli [10,12,13,14,15]. Among lactobacilli, strains ascribed to *Levilactobacillus brevis*, *Lactiplantibacillus plantarum*, and *Fructilactobacillus sanfranciscensis* species are frequently isolated from Italian sourdoughs [16,17,18,19]. In addition, other LAB, such as enterococci, lactococci, pediococci, streptococci, as well as members of the *Leuconostoc* and *Weissella* genera have been detected [4]. Among yeasts, *Saccharomyces cerevisiae, Kazachstania exigua*, *Kazachstania humilis*, *Torulaspora delbrueckii*, *Wickerhamomyces anomalus*, and *Pichia kudriavzevii* commonly dominate the fungal community [20,21,22,23]. During the fermentation process, the yeasts are primarily responsible for leavening whereas the LAB carry out the acidification of the dough and both contribute to the flavor of the resulting bread [21,24]. Although functional and nutritional features of sourdough have been traditionally attributed to the LAB metabolism [25,26], several studies highlighted a link with the activities of the yeast population. Several yeasts, isolated from traditional sourdough samples, exhibited functional properties, such as the ability to produce vitamins [27,28], to exert phytase [29] and antioxidant [30,31] activities as well as probiotic properties [4,23,32,33,34]. 

One of the new trends in bakery productions is the rediscovery of landraces/historical, which is in line with the increased interest in locally grown crops with particular nutritional properties [35]. Compared to modern varieties, wheat landraces have a better nutritional profile, higher content of phenols, vitamins, minerals, and nutraceutical compounds [36]. Sicily, a region of Southern Italy, claims several durum wheat landraces. Among these, *Triticum aestivum* L. subsp. *aestivum*, commonly named Maiorca or Majorca, is one of the durum wheat landraces used in the production of bread, pasta, and cakes until the 1900s. Recently, Maiorca flour was re-discovered and re-employed by local bakers for the distinctive sensory properties generated in the final products [23,36]. As reported by Visioli and co-workers [36], the bread wheat Maiorca showed the highest amount of both high molecular weight and low molecular weight glutenins, compared to other Sicilian durum wheat landraces, such as Margherito, Perciasacchi, Russello, and Timilia. The aforementioned glutenin subunits are important parameters affecting the gluten strength, and therefore, the ability of the proteins to form a tenacious network, promoting extrusion properties. In addition, bread wheat Maiorca exhibited low total gluten proteins [36].

Although the microbial population of many types of sourdough was in-depth investigated, no information is now available about the microbiological profile of the Maiorca sourdough. Therefore, the aim of the present study was to characterize the microbiota of spontaneously fermented Maiorca doughs and to collect detailed information about the composition of both LAB and yeast populations.

## 2. Materials and Methods

### 2.1. Sourdough Sampling 

Sourdough samples (M1, M2, M3, and M4), produced using *Triticum vulgare* Host. var. *albidum* Koern (Maiorca grain), were collected from four artisanal bakeries located in the Sicily region. The characteristics of the sourdough samples are detailed in Table S1. All sourdoughs were propagated by one daily back slopping using the sourdough coming from a previous fermentation without the addition of baker’s yeast or any other starter cultures. Mature sourdough M1, M2, M3, and M4 samples were aseptically collected and transferred to the microbiology laboratory under refrigerated conditions. Three independent samples were taken from each selected bakery from each of three different bread-making runs.

### 2.2. Microbiological Analysis 

Each sourdough sample (25 g) was mixed with 225 mL of sterile saline water (0.9% NaCl *w*/*v*) in a stomacher bag and homogenized for 4 min using a Stomacher (Interscience). Ten-fold serial dilutions were made and plated on the following agar media and conditions: Plate Count Agar (PCA) (Biolife Italiana srl, Milan, Italy), incubated for 48–72 h at 30 ± 2 °C, for total mesophilic bacteria counts; Slanetz Bartley Agar (SLA) (Biolife Italiana srl), incubated for 24–48 h at 37 °C, for enterococci enumeration; Violet Red Bile Glucose Agar (VRBGA) (Biolife Italiana srl), anaerobically incubated at 37 °C for 24–48 h for Enterobacteria; Brilliance E. coli (BEC) (Oxoid, Milan, Italy), incubated at 37 °C for 18–24 h for *Escherichia coli*; Mannitol Salt Agar (MSA) (Biolife Italiana srl), incubated at 32 °C for 48 h, for staphylococci; modified MRS (mMRS), according to Corsetti and co-workers [13], anaerobically incubated at 30 ± 2 °C for 48 h, for lactic acid bacteria; Sabouraud Dextrose Agar (SDA) (Liofilchem, Roseto degli Abruzzi, Italy) and Wallerstein Laboratory Nutrient Agar (WLN) (Liofilchem), supplemented with chloramphenicol (100 mg/L), incubated at 30 ± 2 °C for 48–72 h, for yeasts count. 

### 2.3. Isolation, DNA Extraction and Identification of Lactic Acid Bacteria and Yeasts

From each mMRS agar plate, 20% of the total number of colonies were randomly selected. After purification, by streaking three times onto mMRS agar plates, each colony was microscopically examined, checked for catalase activity and Gram reaction. Isolates were stored at −80 °C in liquid culture using 20% (*v*/*v*) of glycerol until the use. Total genomic DNA was extracted from pure overnight cultures according to the protocol reported by Pino et al. [37]. DNA concentration was assessed by using the Fluorometer Qubit (Invitrogen, Carlsbad, CA, USA). PCR amplification of the 16S rRNA gene was performed using the primer pair 7-f (5′-AGA GTT TGA TC/TA/C TGG CTCAG-3′) and 1510-r (5′-ACG G (C/T) TACC TTG TTA CGA CTT-3′) according to Pino and co-workers [38]. The restriction endonucleases *Alu*I and *Msp*I (Thermo Fisher Scientific, Waltham, MA, USA) were used for the digestion of amplification products as previously reported [39]. PCR products, of at least one strain representative of each restriction pattern, were purified using the QIAquick PCR Purification Kit (Qiagen, Milan Italy) and sequenced on both strands using external primers through a DNA Sanger sequencing process performed by Eurofins Genomics (Vimodrone, Italy). LAB taxonomic identification was assessed by sequence analysis of the 16S ribosomal RNA gene using the the basic local alignment search tool (BLASTn) software in the NCBI-curated bacterial and Archaea RefSeq database. Sequences with a percentage identity ≥98% were considered to belong to the same species. The 16S rRNA gene sequences were deposited in GenBank under the accession numbers from OL314786 to OL314809. 

Presumptive yeasts (20% of the total number of colonies) were randomly selected from both SDA and WLN agar media. Isolates were purified by streaking three times onto the same isolation medium, microscopically examined and stocked at −80 °C in liquid culture using 20% (*w*/*v*) of glycerol. DNA concentration was determined using a Nanodrop Nd 1000 spectrophotometer (260/280 nm) (Nano-drop Technologies, Wilmington, DE, USA). PCR amplification of 5.8S rRNA gene and the upstream and downstream internal transcribed spacer regions ITS1 and ITS2 (globally referred to as ITS regions) was carried out with the primers ITS1 (5′-TCCGTAGGTGAACCTGCGG-3′) and ITS4 (5′-TCCTCCGCTTATTGATATGC-3′) [40]. A 40 μL reaction mixture contained 1X Dream Taq Green Buffer (Mg2^+^ plus) (Thermo Scientific, Waltham, MA, USA), 200 µM of each dNTPs (Thermo Scientific, Waltham, MA, USA), 0.3 µM of each primer, 1 U of Dream Taq DNA polymerase (Thermo Scientific, Waltham, MA, USA) and 50 ng of gDNA template. Amplification reactions were performed in a T100 thermal cycler (BioRad, Hercules, CA, USA) with the following cycling conditions: initial denaturation at 95 °C for 5 min; 35 cycles of denaturation at 95 °C for 1 min, annealing at 55 °C for 2 min, and polymerization at 72 °C for 2 min; the polymerization was finalized by one more cycle at 72 °C for 10 min. ITS amplicons were subjected to RFLP analysis using the endonucleases *Hae*III and *Hinf*I (Thermo Fisher Scientific, Waltham, MA, USA) as reported by Esteve-Zarzoso et al. [41] and according to the manufacturer’s instructions. The restriction fragments were checked by electrophoresis in 2.0% (*w*/*v*) agarose gel. PCR and RFLP fragment lengths were compared with those contained in the database at www.yeast-id.org (CSIC and University of Valencia, Spain) with rank parameters at +/−10. At least one strain representative of each restriction pattern was submitted to PCR amplification of D1/D2 region of the 26S rRNA gene using primers NL1 (5′-GCATATCAATAAGCGGAGGAAAAG-3′) and NL4 (5′-GGTCCGTGTTTCAAGACGG-3′) [42]. The thermal conditions consisted of initial denaturation at 95 °C for 5 min followed by 36 cycles of denaturation at 95 °C for 1 min, annealing at 52 °C for 45 s and extension at 72 °C for 2 min; final extension at 72 °C for 10 min. All PCR products were purified with DNA Clean & Concentrator™-5 Kit (Zymo Research, Orange, CA, USA) and sequenced on both strands using NL1 and NL4 primers by a Sanger method process performed by external service (Bio-Fab Research srl, Rome, Italy). Sequences were edited manually, and consensus sequences were generated using the program SeqMan (DNASTAR, Madison, WI, USA). Sequences were compared against NCBI-RefSeq database for 26S rRNA gene sequences using BLASTn [43]. The sequences of 26S rRNA D1/D2 regions were deposited in GenBank under the accession numbers from MN058031 to MN058041 and from MZ221124 to MZ221135.

### 2.4. Genotyping of Lactic Acid Bacteria and Yeasts

All the LAB and yeast isolates were genotyped with a repetitive element palindromic PCR (rep-PCR) fingerprinting analysis with (GTG)_5_ primer (5′-GTG GTG GTG GTG GTG-3′) [44], according to the protocols reported in Solieri et al. [45] and Dakal et al. [46], respectively. Briefly, both PCR amplification reactions of LAB and yeasts isolates were carried out in a 20 µL final volume containing 1 x DreamTaq Green Buffer, 3 mM MgCl_2_, 200 µM of each dNTP, 5 U of DreamTaq DNA polymerase, 50 ng of template DNA and either 0.6 µM or 1 µM of (GTG)_5_ primer for yeast and LAB, respectively. In addition, the PCR mixture for LAB genotyping contained 100 µg/mL of BSA (bovine serum albumin) (Thermo Fisher Scientific, Waltham, MA, USA). All rep-PCR reactions were carried out in the same T100™ thermal cycler, using the following cycling parameters: for LAB, initial denaturation at 94 °C for 5 min; 35 cycles of denaturation at 94 °C for 30 s, annealing at 40 °C for 1 min, and extension at 72 °C for 4 min; final extension at 72 °C for 7 min; for yeasts, initial denaturation at 94 °C for 5 min; 40 cycles of denaturation at 94 °C for 15 s, annealing at 55 °C for 45 min, and extension at 72 °C for 1.30 min; final extension at 72 °C for 4 min. The products were run on 1.8% (*w*/*v*) agarose gel containing SYBR™ Safe DNA Gel Stain (Invitrogen, Waltman, MA, USA) for 6 h at the constant voltage of 70 V in 0.5X TBE buffer under refrigerated conditions. GeneRuler 100 bp Plus DNA Ladder (Thermo Fisher Scientific, Waltham, MA, USA) was used as molecular-weight standard. The obtained digitalized images were analyzed using the BioNumerics software v3.0 (Applied Maths, Sint-Martens-Latem, Belgium). Pearson’s correlation similarity coefficient was used to convert computed band patterns into a similarity matrix. Optimization and curve smoothening parameters were optimized with the specific script present in BioNumerics. The unweighted pair group method analysis using the arithmetic means (UPGMA) was used for the tree construction. LAB and yeast isolates of ≥80.2 and 92% similarity values were treated as a single strain, respectively. Reproducibility cut-off values were established in at least two PCR amplification reactions using the same DNA preparation from three randomly selected LAB or yeast strains.

### 2.5. Statistical and Phylogenetic Analysis

All experiments were performed in triplicate and data were reported as average values, provided with standard deviation. Data were subjected to one-way ANOVA and pair-comparisons were achieved by Tukey’s procedure at *p* < 0.05, using the Minitab 19 Statistical software. 

Regarding LAB and yeasts phylogenetics, the related sequences were aligned with Muscle program [47] in MEGA X software [48] and the resulting alignment was subjected to a DNA substitution model analysis to select the best-fitting model. Phylogenetic relationships were computed using the Kimura 2-parameter (K2P) model and the Neighbor Joining (NJ) method, whereas a gamma distribution (+G) was used to model rate variation among sites. Bootstrap support values were obtained from 1000 random re-samplings. Interactive Tree of Life (ITOL) was used to visualize all trees [49].

## 3. Results

### 3.1. Microbiological Analysis 

Results of the main microbial groups detected in M1, M2, M3, and M4 sourdough samples are reported in Table 1. Overall, high variability was observed among samples except for Enterobacteriaceae, Staphylococcus spp., and Escherichia coli were never detected. In detail, the highest mesophilic aerobic bacteria population was detected in the M1 sample whereas M3 and M4 samples exhibited the highest enterococci count. Lactic acid bacteria were detected in all samples with an average value of 8.59 log CFU/g. The M2 sample exhibited the highest LABs cell density (9.14 log CFU/g) whereas the lowest was observed in the M1 sample (7.88 log CFU/g). The yeast population showed a cell density of about 8 log CFU/g in all analyzed samples except for the M2 sample which was characterized by a lower count (6.73 log CFU/g) (Table 1).

### 3.2. LAB identification and Species Distribution

**Figure 1:**
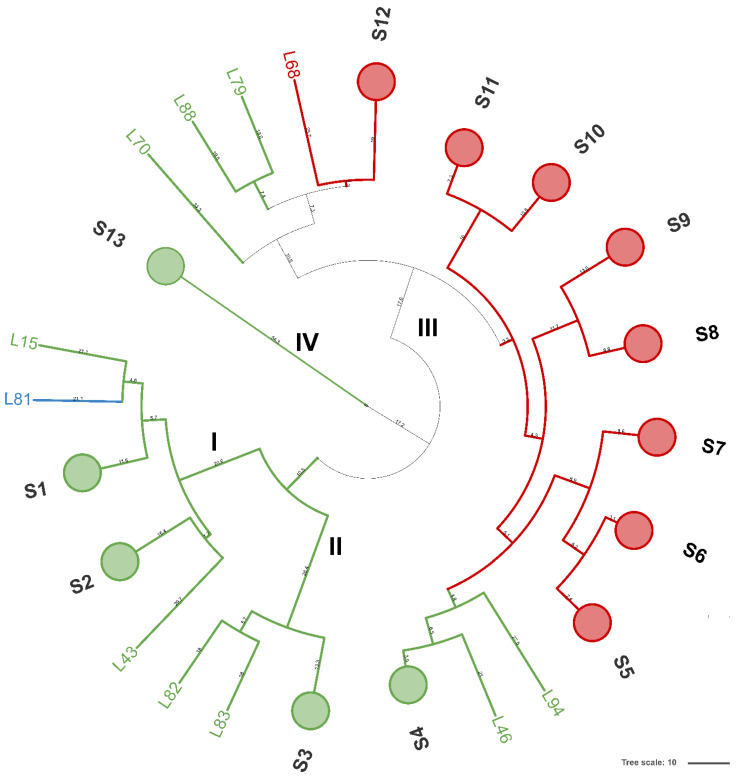
Dendrogram analysis of (GTG)_5_-based rep-PCR patterns of LAB isolates. Similarities were calculated as Pearson r product-moment correlation coefficient and dendrogram was built using the Unweighted Pair Group Method with Arithmetic Mean (UPGMA). Numbers near the branch represent branch length. Circles are subclusters of biotypes obtained with a reproducibility cut-off of 80.2% and are labeled from S1 to S13. Major clusters are numbered with Roman letters from I to IV. Cluster and/or singletons belonging to *L. brevis*, *L. plantarum*, and *L. rhamnosus* are reported in red, green, and light blue, respectively. The tree was visualized using Itol.

**Figure 2:**
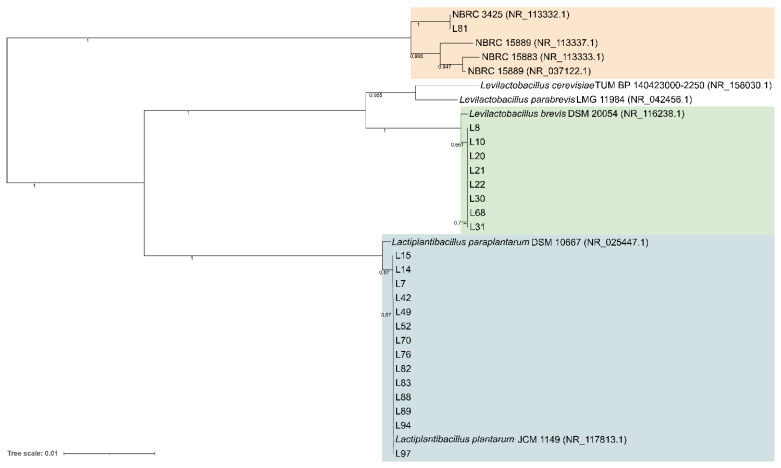
Neighbor-joining phylogeny of LAB strains based on 16S rRNA gene partial sequences. Bootstrap supports (1000 replicates) >0.4 are shown next to the branches. The evolutionary distances were computed using the Kimura 2-parameter and the rate variation among sites was modeled with a gamma distribution (shape parameter = 1). The tree is drawn to scale, with branch lengths measured in the number of base substitutions per site. The analysis involved 32 nucleotide sequences. All ambiguous positions were removed for each sequence pair. There were a total of 1436 positions in the final dataset.

**Figure 3:**
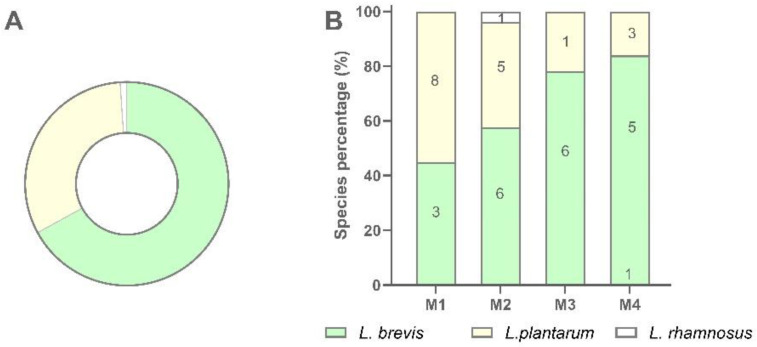
Recovery percentage and species distribution of LAB isolates in sourdough samples. (**A**) Percentage of recovery of each LAB species; (**B**) Distribution of each species in M1, M2, M3, and M4 sourdough samples.

A total of 94 LAB isolates were preliminarily characterized by 16S rRNA gene ARDRA analysis. Simultaneous double digestion with the restriction enzymes *Alu*I and *Msp*I allowed for the discrimination of isolates into three different restriction patterns, namely A, B, and C which were congruent with those expected for the species *Levilactobacillus brevis*, *Lactiplantibacillus plantarum*, and those of the *Lacticaseibacillus casei* group, respectively (supplementary Table S2). rep-PCR fingerprinting with (GTG)_5_ primer was performed to genotype the library of LAB isolates. (GTG)_5_ fingerprints consisted of band numbers from 5 to 19 and band sizes ranging from 280 to 2630 bp. Clustering analysis of (GTG)_5_-fingerprints showed that four major clusters, named from I to IV, were found at the similarity threshold of 50% (Figure 1). Cluster I mainly grouped 13 isolates with 16S rRNA gene ARDRA pattern B and one isolate with pattern C, whereas cluster II consisted of five isolates most of which showed 16S rRNA gene ARDRA profile A. Cluster III was an intermixed group of 71 isolates with patterns A and B, whilst cluster IV grouped five isolates showing the restriction profile B. Using 80.2% as reproducibility threshold we grouped 83 isolates into 13 subclusters and identified 11 singletons. This similarity cut-off was chosen from duplicate experiments to estimate the experimental reproducibility of (GTG)_5_ typing method (data not shown). At least one strain representative of each subcluster and/or strains for which incongruence was scored between (GTG)_5_ clustering and 16S rRNA gene ARDRA profile was submitted to the sequencing of the 16S rRNA gene and BLASTn analysis (Table S3). As reported in Figure 2, an NJ-based tree was built to identify the phylogenetic relationships among isolates. Strains with 16S rRNA gene ARDRA profile B formed a monophyletic and statistically supported clade with *L. plantarum* JCM1149, indicating their affiliation to this species. Strains with 16S rRNA gene ARDRA profile A clustered with the type strain of *L. brevis* DSM 20054^T^, while strain L81 clustered with *L. rhamnosus* NBR3425 with 100% bootstrapping (Figure 2). Congruently with the species attribution, eight subclusters, namely S1, S2, S3, S4, and S13, grouped 21 *L. plantarum* strains, while eight *L. plantarum* isolates and *L. rhamnosus* L81 were singletons. Sixty-four *L. brevis* isolates grouped into eight subclusters within the major cluster III, namely from S5 to S12, while L68 and L79 were the only two *L. brevis* singletons. Strain L14 belonged to *L. plantarum* but incongruently clustered into S5 together with *L. brevis* isolates (Table S3). This evidence supported that (GTG)_5_ rep-PCR should be integrated with other techniques, such as 16S rRNA gene ARDRA to properly select strains for sequencing and to avoid any misidentifications. Figure 3 shows that *L. brevis* and *L. plantarum* were scored in all the samples but with different percentages. In particular, *L. plantarum* was the dominant species in sample M1, while *L. brevis* dominated M2, M3, and M4 samples. *L. rhamnosus* was scored only in sample M2. The number of genotypes was high for every species in all the samples suggesting a high diversity in LAB communities populating these sourdoughs.

### 3.3. Yeast Identification and Species Distribution

**Figure 4:**
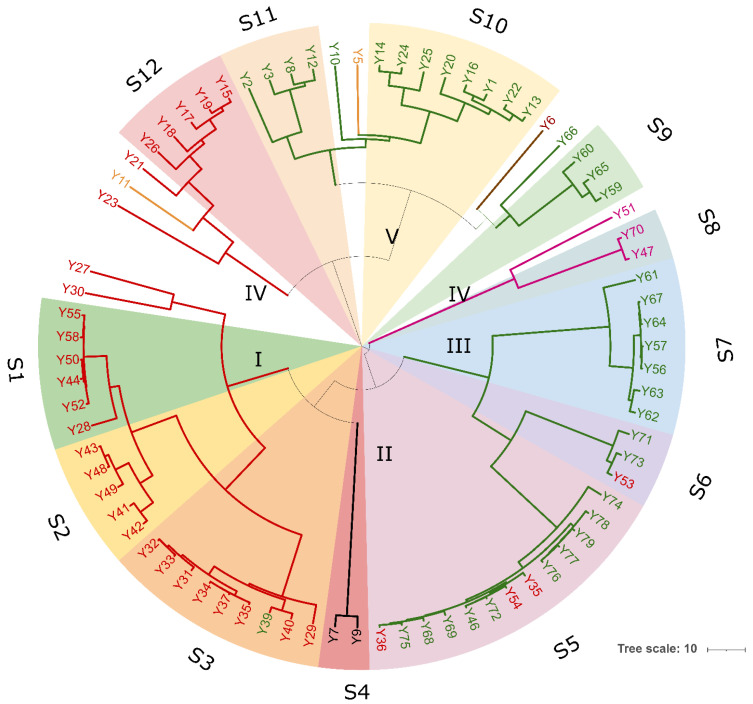
Clustering analysis of (GTG)_5_-based rep-PCR patterns of yeast isolates. Similarities were calculated as Pearson r product-moment correlation coefficient and dendrogram was built using the Unweighted Pair Group Method with Arithmetic Mean (UPGMA) method. Major clusters are identified at 50% of similarity and numbered with Roman letters from I to IV. Biotypes and/or singletons obtained with a reproducibility cut-off of 92% are labeled from S1 to S12 and drawn with the following colour code: *S. cerevisiae*, red; *W. anomalus*, green; *P. kluyveri*, black; *T. delbrueckii*, purple; *C. diddensiae*, brown; and *C. boidinii*, orange. The tree was visualized using Itol.

A total of 77 yeast isolates were successfully submitted to PCR amplification of the ITS region. Thirty-seven isolates showed an ITS amplicon of 660 bp, 32 isolates one of 880 bp, while five amplicons of 800, 700, 630, and 450 bp were found for 3, 1, 2, and 2 isolates, respectively. Combination of the ITS amplicons size and the restriction profiles obtained with endonucleases *Hae*III and *Hinf*I allowed the identification of seven different restriction patterns (referred to as A to F) corresponding to *Wickerhamomyces anomalus* (100%), *Saccharomyces cerevisiae* (100%), *Torulaspora delbrueckii* (100%), *Candida boidinii* (73%), *Candida ethanolica* (83%), and *Candida maritima*/*Candida zeylanoides*/*Candida diddensiae* (100%) in yeast-id database, respectively. Digestion of 620 bp amplicons with *Hae*III resulted in undigested fragments in all 37 isolates, while digestion with *Hinf*I produced two fragments of 310 and 300 bp (Table S4). Using *Hae*III and *Hinf*I all the 32 isolates having an 880 bp long fragment resulted in the same restriction patterns (325, 230, 170, and 125 bp with *Hae*III and 375, 365, and 110 bp for *Hinf*I, respectively) as the reference strains *S. cerevisiae* CBS1171^T^. Conversely, two isolates showing 630 bp long ITS amplicon, exhibited pattern F consisting in 420, 130 and 80 bp long fragments with *Hae*III and two fragments of approximatively 310 bp with *Hinf*I, respectively. This pattern did not allow for discrimination between *C. maritima*, *C. zeylenoides*, and *C. diddensiae*. Isolates producing ITS amplicons of 800, 700, and 450 bp length exhibited three distinct restriction patterns (Table S4). The BLASTn searches of 26S D1/D2 region sequences against the GenBank database confirmed species attribution for all the restriction patterns, except for pattern E, which corresponded to *Pichia kluyveri* instead of *C. ethanolica* (Table S5). Phylogenetic analysis showed that all the *S. cerevisiae* strains formed a monophyletic group with *S. cerevisiae* NRRL Y-12632 confirming species attribution based on PCR-RFLP analysis of ITS regions (Figure 4). Strains Y56, Y59, Y60, Y67, and Y1 clustered to *W. anomalus* with a high bootstrapping, forming a taxonomically congruent group within the Debaryomycetaceae clade. Strains Y47, Y70, and Y51 (pattern C) were phylogenetically identical to *T. delbrueckii*, while isolates Y6 representative for pattern F grouped to *C. diddensiae* (90% bootstrapping) (Table S4). Isolates Y5 and Y7 showing patterns D and E respectively, were close to *C. boidinii* and *P. kluyveri* species (Figure 4).

**Figure 5:**
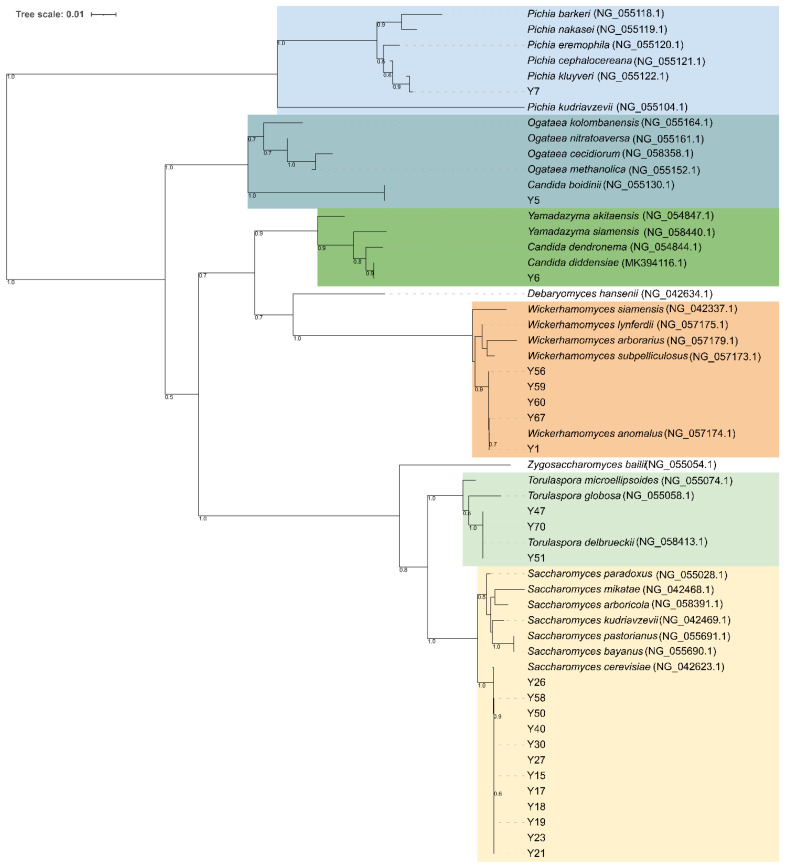
Neighbor-joining phylogeny of yeast strains based on 26S rRNA gene partial sequences. Bootstrap supports (1000 replicates) > 0.5 are shown next to the branches. The evolutionary distances were computed using the Kimura 2-parameter and the rate variation among sites was modeled with a gamma distribution (shape parameter = 1). The tree is drawn to scale, with branch lengths measured in the number of base substitutions per site. The analysis involved 55 nucleotide sequences. All ambiguous positions were removed for each sequence pair. There was a total of 581 positions in the final dataset.

Yeast isolates were genotyped with (GTG)_5_-based rep-PCR. The different band patterns generated by this method ranged from a minimum of six to a maximum of 12 bands, and sizes of amplified fragments between 274 and 2630 bp. The UPGMA dendrogram obtained from the cluster analyses of (GTG)_5_ fingerprints with the Pearson’s similarity coefficient grouped 77 yeast isolates into six major clusters (named from I to VI) at the cut-out value of 60% (Figure 5). Except for some strains, UPGMA cluster analysis provided a differentiation of the isolates according to the species attribution based on ITS PCR-RFLP analysis and 26S D1/D2 domains sequence-based phylogeny. Cluster I and II were homogeneous in species composition and grouped 23 *S. cerevisiae* and two *P. kluyveri* isolates, respectively (Figure 5). Cluster III was heterogeneous in species composition as it included 19 *W. anomalus* and four *S. cerevisiae* isolates, while cluster IV grouped only three isolates one belonging to *S. cerevisiae* and two to T. delbrueckii. Most of *W. anomalus* isolates grouped within cluster V, except for one *C. diddensiae* and one *C. boidinii* strains. Cluster VI was formed by seven *S. cerevisiae* strains and one *C. diddensiae* isolate (Figure 5). When a similarity value of 92% was applied as a reproducibility cut-off, 12 genotypes and 11 singletons strains were scored, suggesting a high level of intra-strain diversity among yeast isolates. We scored 10 biotypes for *S. cerevisiae* and *W. anomalus*, respectively; two for *T. delbrueckii*, two for *C. diddensiae* and one for *P. kluyveri*. 

**Figure 6:**
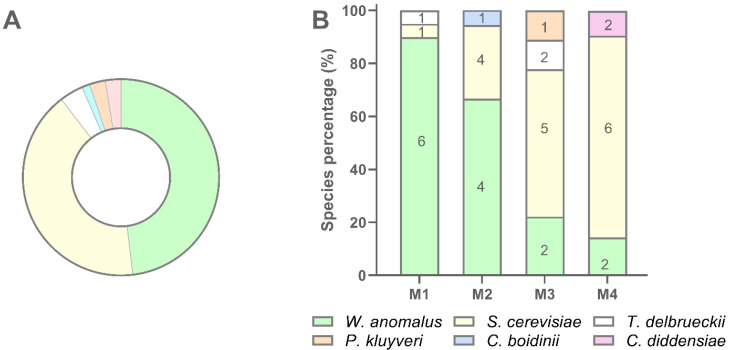
Recovery percentage and species distribution of yeast isolates in sourdough samples. (**A**) Percentage of recovery of each yeast species; (**B**) Distribution of each species in M1, M2, M3, and M4 sourdough samples.

Globally *W. anomalus* accounted for 48.1% of isolates, followed by *S. cerevisiae* (41.6%), *T. delbrueckii* (3.9%), *C. diddensiae* (2.6%), *P. kluyveri* (2.6%), and *C. boidinii* (1.3%) (Figure 6, panel A). *W. anomalus* dominated sample M1 while *S. cerevisiae* was the dominant species in sample M4. Samples M2 and M3 showed *W. anomalus* and *S. cerevisiae* at intermediated distribution compared with M1 and M4 (Figure 6, panel B). In particular, M3 exhibited the most complex species composition as four species, namely *W. anomalus*, *S. cerevisiae*, *T. delbrueckii*, and *P. kluyveri* were recovered in this sourdough. In every sample, a high number of biotypes was found to dominate the yeast population, even if the number of biotypes per sample decreased with the decreasing of the number of isolates (Figure 6, panel B). In several cases, the same biotype was scored into different samples. *C. boidinii* and *C. diddensiae* were scored in only one sample, M2 and M4 respectively, and seemed to be occasional contaminant microorganisms for the low number of isolates recovered (Figure 6).

## 4. Discussion

Sourdough is one of the oldest starters traditionally used for making baked goods. Its use, alternatively to baker’s yeast and chemical leavening, offers several advantages since sourdough has been claimed as able to improve both sensory and rheology of the final product as well as shelf life. Recent data revealed that sourdough fermentation could increase mineral bioavailability, reduce the glycaemic index, improve protein digestibility, and decrease the anti-nutritional factors content [50]. Recently, the increasing attention to organic and natural products promoted the rediscovery of landrace/historical species of wheat. The renewed interest in ancient grains is correlated to the recognition of ancient wheat as a healthy cereal recommended for the treatment of several diseases, such as hypercholesterolemia, colitis, allergies, and insulin resistance [13,23,36]. Among these, *Triticum aestivum* L. subsp. *aestivum*, commonly named Maiorca or Majorca, is one of the Sicilian durum wheat landraces recently re-discovered by both consumers and local bakers. 

Up to now, the microbial population of several traditional sourdoughs has been characterized although, based on our knowledge, no study has been conducted on Maiorca sourdough produced in Sicily. According to that, the present study aimed to characterize the native microbiota of spontaneously fermented Maiorca dough samples, obtained from four different bakeries located in Sicily.

Microbiological data revealed a quite similar cell density for both LAB and yeast populations suggesting the absence of competitiveness between the two communities. Although several studies reported higher LAB counts compared to yeasts one [10,51,52], the ability of both LAB and yeasts to co-dominate the sourdough ecosystems, creating a stable association, is renowned [24]. In the present study, the in-depth characterization of the lactobacilli population revealed that *Lactiplantibacillus plantarum* and *Levilactobacillus brevis* unquestionably dominated the Maiorca sourdough ecosystem. As recently revised by Arora and co-workers [53] the aforementioned species were commonly identified in more than 15 sourdoughs worldwide, meaning that they are the most common representatives of the LAB community. In particular, the dominance of *L. plantarum* was observed in Sardinian [54], Apulian [55], Molisan [56], and Sicilian traditional sourdoughs [16,57]. Similarly, Alfonzo and co-workers [11], by studying the microbial composition of flour samples, used in Sicily to make bread, reported the dominance of the *L. plantarum* species. In addition, Ventimiglia and co-workers [11] highlighted the codominance of *L. plantarum* with obligate heterofermentative species. According to that, the high occurrence of *L. plantarum* could be due to the bread-making technologies applied in Italian sourdough rather than the type of flour, as previously reported [51,58]. The robustness of *L. plantarum* strains, recovered in the sourdoughs analyzed in this study, could be due to several factors, such as rapid acidification, competition for nutrients, adaptability to environmental stresses, as well as ability to ferment hexose and pentose, synthesize diacetyl and hydrogen peroxide, and produce bacteriocins [59]. Similarly, to artisanal sourdough samples, obtained from bakeries located in Northern Italy [60,61], as well as French sourdoughs [62], the co-dominance of *L. plantarum* and *L. brevis* species was found in samples analyzed in the present study. This could be attributed to low incubation temperatures and continuous back-slopping creating a favorable environment for the development of the aforementioned species, as reported by De Vuyst and co-workers [4]. In discordance to previously reported data, which highlighted that *Fructilactobacillus sanfranciscensis* and *Limosilactobacillus fermentum* were frequently isolated from Italian and European sourdoughs [16,53,57,63], the aforementioned species were not detected in the analyzed Maiorca sourdough samples. According to that, Gobbetti and co-workers [64] reported on the strict association between *L. sanfranciscensis* and *L. plantarum* in Italian wheat sourdoughs and Minervini and co-workers [65] demonstrated the robustness of the *L. plantarum* under daily back-slopping propagation using wheat flour. 

Concerning yeasts community, the rep-PCR with (GTG)_5_ primer method has been demonstrated to be powerful for discriminating biotypes in the yeast sourdough population [66] However, Ramírez-Castrillón et al. [67] proved that fingerprinting analysis carried out with only one microsatellite primers can group isolates belonging to different species in the same cluster leading to misidentification. We also found incongruences between species attribution carried out with conventional DNA barcoding approaches, such as ITS PCR-RFLP and 26S D1/D2 rDNA sequencing, and clustering results obtained by DNA fingerprinting with the (GTG)_5_ primed rep-PCR. These results supported that a combination of different molecular approaches is required for proper species attribution and intra-specific polymorphism detection of sourdough yeasts isolates. Compared to extensive sourdoughs studies [10,68,69,70], high species diversity was found albeit only four bakeries were sampled. *Saccharomyces cerevisiae* and *Wickerhamomyces anomalus* were the most frequently isolated species. It is well known that both *S. cerevisiae* and *W. anomalus* species are characterized by high adaptability to stressful conditions in terms of temperature, pH and osmolarity [24,69,70,71,72,73]. In particular, strains ascribed to the *S. cerevisiae* species are frequently isolated in sourdoughs from central and southern Italy [10,21,23]. *S. cerevisiae* is the yeast most frequently present in fermented sourdough, as it is able to produce a significant amount of ethanol in sourdoughs by consuming both maltose and glucose [74]. It was interesting to point out that *L. brevis* was recovered with a high percentage of isolation in samples dominated by *S. cerevisiae*. This result is in discordance to previously reported data, demonstrating the greatest adaptability of *S. cerevisiae* when it is associated with homofermentative compared to heterofermentative LAB [60,75]. In addition, the presence of both *Candida boidinii* and *L. brevis*, revealed in the M2 sample, could be associated with the absence of competition for maltose. As previously reported by Solieri and co-workers [76], *Candida* spp. ferments glucose and trehalose but not maltose. The high frequency of isolates ascribed to the *W. anomalus* species corroborates its well-known ability to thrive in a wide range of microbial ecosystems thanks to its competitiveness under stressful environmental conditions [71]. Congruently to the absence of *L. sanfranciscensis*, we also did not observe the maltose negative *K. humilis* in the yeast community. The stable association between maltose positive and maltose negative *K. humilis* due to the lack of antagonism for maltose has been stated by many authors [15,77]. The absence of both *L. sanfranciscensis* and *K. humilis*, in the analyzed Maiorca sourdough samples, could be probably related to external conditions, such as the environmental temperature, occurring during the sourdough fermentation [77,78].

*Torulaspora delbrueckii* and *P. kudriavzevii* have been recovered among the six yeast species most frequently associated with sourdoughs [24]. Congruently these species were scored in the analyzed Maiorca sourdoughs even if at low frequency. *T. delbrueckii* is recognized as a superior choice due to its importance in the production of frozen dough products since it exhibits a very good baking ability and a high capacity to resist osmotic and freeze–thaw stresses [79]. According to that, *T. delbrueckii* has already been used in the bakery industry in Japan, for the production of sweetbreads and pastries. *P. kudriavzevii* was found as a non-dominant yeast species in one out of four Danish sourdoughs in association with *K. exigua*, while *P. kudriavzevii* was found as a pure yeast culture in two Lithuanian sourdoughs. Only a few studies have, as yet, reported on the isolation of *P. kudriavzevii* from artisan Belgian [69] and Chinese [80] sourdoughs.

## 5. Conclusions

The culture-dependent approach, applied in the present study, allowed for in-depth investigation, for the first time, of the LAB and yeasts population of Sicilian Maiorca sourdough samples. LAB and yeasts co-dominated the sourdough ecosystem of the analyzed samples, creating a stable association. Among LAB, *Lactiplantibacillus plantarum* and *Levilactobacillus brevis* dominated the Maiorca sourdough ecosystem, whereas high species diversity was found among yeasts. Further studies will be conducted in order to evaluate both the pro-technological and functional properties of the isolated strains to be used as starters for the production of baked goods.

## Figures and Tables

**Table 1:** Microbiological counts expressed as log_10_ CFU/g ± standard deviation of the main microbial groups detected in M1, M2, M3, and M4 sourdough samples collected from four different Sicilian bakeries.

Samples	Mesophilic Aerobic Bacteria	LAB	*Enterococcus* spp.	Yeasts
M1	9.42 ^a^ ± 0.04	7.88 ^d^ ± 0.04	3.72 ^b^ ± 0.05	8.29 ^a^ ± 0.02
M2	6.17 ^d^ ± 0.03	9.14 ^a^ ± 0.02	2.52 ^c^ ± 0.05	6.73 ^c^ ± 0.03
M3	7.32 ^c^ ± 0.03	8.52 ^c^ ± 0.01	3.98 ^a^ ± 0.08	8.28 ^a^ ± 0.03
M4	8.61 ^b^ ± 0.02	8.81 ^b^ ± 0.02	3.91 ^a^ ± 0.02	8.17 ^b^ ± 0.03

^a–d^: for each microbial group, data in the same column with different superscript letters are significantly different (*p* < 0.05).

## Data Availability

The data presented in this study are included within the article.

## Supplementary Materials

The following supporting information can be downloaded at: https://www.mdpi.com/article/10.3390/microorganisms10020283/s1, Table S1. Characteristics of the sourdough samples M1, M2, M3, and M4 collected from the four selected bakeries located in Sicily. Table S2. Overview of the 16S rRNA gene ARDRA profiles and clustering data based on (GTG)_5_ rep-PCR. Table S3. 16S rRNA gene sequencing results and BLAST search on NCBI RefSeq database (16S ribosomal RNA nucleotide sequence records for Bacteria and Archaea). Table S4. Overview of the ITS restriction patterns of sourdough yeast isolates. Table S5. 26S rRNA D1/D2 region sequencing results and BLAST search on NCBI RefSeq database (28S ribosomal RNA Nucleotide sequence records).
