# Supplementary material for: Microbial Consortia Involved in Traditional Sicilian Sourdough: Characterization of Lactic Acid Bacteria and Yeast Populations
Source: Microorganisms. 2022 Jan 26;10(2):283. doi: 10.3390/microorganisms10020283 (PMC8875953; doi:10.3390/microorganisms10020283)
Supplement: Supplementary file 1 [file microorganisms-10-00283-s001.zip › microorganisms-1533816-supplementary.pdf]

**Table S1.** Characteristics of the sourdough samples M1, M2, M3, and M4 collected from the four selected bakeries located in Sicily.

| | M1 | M2 | M3 | M4 |
|---|---|---|---|---|
| Bakery location (city) | Maletto | Trapani | Palermo | Catania |
| Flour in chief sourdough | Maiorca | Maiorca | Maiorca | Maiorca |
| Hydration of sourdough (%) | 87 | 75 | 82 | 73 |
| No. of back-slopping steps before use | 1 | 1 | 1 | 1 |
| Temp (°C) of back-slopping[a] | 22.5 | 21 | 23 | 22.6 |
| Period (h) of back-slopping[a] | 6h30 | 6h | 11h | 14h |
| Sourdough[b] (%) | 43 | 16 | 15.1 | 33 |
| Flour[b] (%) | 37 | 44 | 49.4 | 39.4 |
| Water[b] (%) | 19 | 38 | 35 | 27 |
| Salt[b] (%) | 0.9 | 1.3 | 0.8 | 0.8 |
| Kneading method[c] | Me | Me | Ma | Me |
| Total period of fermentation | 4h30 | 6h50 | 4h45 | 4h20 |

[a] Mature sourdough, after the last back-slopping
[b] Percentage of total dough weight
[c] Mechanically (Me) or manually (Ma)
[d] For the second bread-making run, only wheat flour was used

**Table S2.** Overview of the 16S rRNA gene ARDRA profiles and clustering data based on $(GTG)_5$ rep-PCR.

| Samples | Strains | 16S ARDRA analysis | | $(GTG)_5$ rep-PCR | |
|---|---|---|---|---|---|
| | | Restriction fragments (bp) | Pattern | Subcluster | Major cluster |
| M1 | **L7** | 313-305-246-110-100-90 | B | S1 | I |
| | L84, **L89** | 313-305-246-110-100-90 | B | S2 | I |
| | **L82** | 313-305-246-110-100-90 | B | Singleton | II |
| | **L83** | 313-305-246-110-100-90 | B | Singleton | II |
| | L92, L96, **L97** | 313-305-246-110-100-90 | B | S3 | II |
| | L90 | 313-305-246-110-100-90 | B | S4 | III |
| | **L94** | 313-305-246-110-100-90 | B | Singleton | III |
| | **L88** | 313-305-246-110-100-90 | B | Singleton | III |
| | L5, L6, L93 | 510-240-110-105-90 | A | S5 | III |
| | L4, L91, L95 | 510-240-110-105-90 | A | S7 | III |
| | L1, L2, L3 | 510-240-110-105-90 | A | S9 | III |
| M2 | **L10,** L64, L69, L67, L77, L9, L11, **L14, L8,** L66 | 510-240-110-105-90 | A | S5 | III |
| | L74 | 510-240-110-105-90 | A | S8 | III |
| | L12, L13 | 510-240-110-105-90 | A | S9 | III |
| | L65 | 510-240-110-105-90 | A | S10 | III |
| | **L68** | 510-240-110-105-90 | A | Singleton | III |
| | **L79** | 510-240-110-105-90 | A | Singleton | III |
| | L73, **L76,** L78, L80, L72 | 313-305-246-110-100-90 | B | S1 | I |
| | **L15** | 313-305-246-110-100-90 | B | Singleton | I |
| | L75 L71 | 313-305-246-110-100-90 | B | S2 | I |
| | **L81** | 310-310-220-212-115-90 | C | Singleton | I |
| | **L70** | 313-305-246-110-100-90 | B | Singleton | III |
| M3 | L50, L55, L56, **L49, L52** | 313-305-246-110-100-90 | B | S13 | IV |
| | **L21, L22,** L19, **L20,** L16, L17, L23 | 510-240-110-105-90 | A | S5 | III |
| | L51 | 510-240-110-105-90 | A | S7 | III |
| | L59 | 510-240-110-105-90 | A | S8 | III |
| | L18 | 510-240-110-105-90 | A | S9 | III |
| | L58, L63, L53, L54, L61 | 510-240-110-105-90 | A | S10 | III |
| | L57, L62, L60 | 510-240-110-105-90 | A | S11 | III |
| M4 | L43 | 313-305-246-110-100-90 | B | Singleton | I |
| | **L42,** L44 | 313-305-246-110-100-90 | B | S4 | III |
| | L46 | 313-305-246-110-100-90 | B | Singleton | III |
| | **L30,** L28, L29, L27, L26, L33, L38, L37, L39, L24, L25 | 510-240-110-105-91 | A | S5 | III |
| | L36, L47, L45, L35, | 510-240-110-105-91 | A | S6 | III |
| | L40, L41 | 510-240-110-105-91 | A | S7 | III |

| | | | | |
|---|---|---|---|---|
| **L31** | 510-240-110-105-91 | A | S11 | III |
| L32, L34, L48 | 510-240-110-105-91 | A | S12 | III |

Restriction fragments were determined by simultaneously cutting 1500bp-long 16S rRNA gene amplicons with the endonucleases *Alu*I and *Msp*I. Fragments lower than 70 bp were omitted from the restriction analysis. Major clusters and subclusters were defined through $(GTG)_5$ rep-PCR analysis using 50 and 80.2% of similarity as cut-off values, respectively. Strains in bold were submitted to 16S rRNA gene sequencing.

**Table S3.** 16S rRNA gene sequencing results and BLAST search on NCBI RefSeq database (16S ribosomal RNA nucleotide sequence records for Bacteria and Archaea).

| Sample | Strain code | Accession number | BLASTn search best hit | | | | |
|---|---|---|---|---|---|---|---|
| | | | Max Score | Query cover (%) | *E*-value | Identity (%) | Description |
| M1 | L7 | OL314786 | 2651 | 100 | 0.0 | 100 | *L. plantarum* NRRL B-14768 (NR_104573.1) |
| | L89 | OL314807 | 2651 | 100 | 0.0 | 100 | *L. plantarum* NRRL B-14768 (NR_104573.1) |
| | L82 | OL314804 | 2651 | 100 | 0.0 | 100 | *L. plantarum* NRRL B-14768 (NR_104573.1) |
| | L83 | OL314803 | 2651 | 100 | 0.0 | 100 | *L. plantarum* NRRL B-14768 (NR_104573.1) |
| | L97 | OL314809 | 2651 | 100 | 0.0 | 100 | *L. plantarum* NRRL B-14768 (NR_104573.1) |
| | L94 | OL314808 | 2651 | 100 | 0.0 | 100 | *L. plantarum* NRRL B-14768 (NR_104573.1) |
| | L88 | OL314806 | 2651 | 100 | 0.0 | 100 | *L. plantarum* NRRL B-14768 (NR_104573.1) |
| M2 | L76 | OL314801 | 2651 | 100 | 0.0 | 100 | *L. plantarum* NRRL B-14768 (NR_104573.1) |
| | L15 | OL314790 | 2651 | 100 | 0.0 | 100 | *L. plantarum* NRRL B-14768 (NR_104573.1) |
| | L81 | OL314803 | 2761 | 100 | 0.0 | 100 | *L. rhamnosus* NBRC 3525(NR_113332.1) |
| | L10 | OL314788 | 2604 | 99 | 0.0 | 99.86 | *L. brevis* DSM20054 (NR_116238.1) |
| | L14 | OL314789 | 2651 | 100 | 0.0 | 100 | *L. plantarum* NRRL B-14768 (NR_104573.1) |
| | L68 | OL314799 | 2604 | 99 | 0.0 | 99.86 | *L. brevis* DSM20054 (NR_116238.1) |
| | L79 | OL314802 | 2604 | 99 | 0.0 | 99.86 | *L. brevis* DSM20054 (NR_116238.1) |
| | L70 | OL314800 | 2651 | 100 | 0.0 | 100 | *L. plantarum* NRRL B-14768 (NR_104573.1) |
| M3 | L21 | OL314792 | 2604 | 99 | 0.0 | 99.86 | *L. brevis* DSM20054 (NR_116238.1) |
| | L22 | OL314793 | 2604 | 99 | 0.0 | 99.86 | *L. brevis* DSM20054 (NR_116238.1) |
| | L20 | OL314791 | 2604 | 99 | 0.0 | 99.86 | *L. brevis* DSM20054 (NR_116238.1) |
| | L49 | OL314797 | 2651 | 100 | 0.0 | 100 | *L. plantarum* NRRL B-14768 (NR_104573.1) |
| | L52 | OL314798 | 2651 | 100 | 0.0 | 100 | *L. plantarum* NRRL B-14768 (NR_104573.1) |
| M4 | L42 | OL314796 | 2651 | 100 | 0.0 | 100 | *L. plantarum* NRRL B-14768 (NR_104573.1) |
| | L30 | OL314794 | 2604 | 99 | 0.0 | 99.86 | *L. brevis* DSM20054 (NR_116238.1) |
| | L31 | OL314795 | 2604 | 99 | 0.0 | 99.86 | *L. brevis* DSM20054 (NR_116238.1) |

**Table S4**. Overview of the ITS restriction patterns of sourdough yeast isolates.

| Sample | Strain | Amplicon | Restriction profile | | Yeast-id Best matching | Pattern code |
|---|---|---|---|---|---|---|
| | | | *Hae*III | *Hinf*I | | |
| M1 | Y1, Y2, Y3, Y8, Y12, Y66, **Y67,** Y68, Y69, Y71, Y72, Y73, Y74, Y75, Y76, Y77, Y78, Y79 | 620 | 620 | 310-300 | *W. anomalus* (100%) | A |
| | **Y23** | 880 | 325-230-170-125 | 375-365-110 | *S. cerevisiae* (100%) | B |
| | Y70 | 800 | 800 | 410-380 | *T. delbrueckii* (100%) | C |
| M2 | Y53, Y54, Y55, **Y58, Y26** | 880 | 325-230-170-125 | 375-365-110 | *S. cerevisiae* (100%) | B |
| | Y10, Y13, Y22, Y56, Y57, Y59, **Y60,** Y61, Y62, Y63, Y64, Y65 | 620 | 620 | 310-300 | *W. anomalus* (100%) | A |
| | **Y5** | 700 | 700 | 320-200-150 | *C. boidinii* (73%) | D |
| M3 | Y14, Y24, Y25, Y46 | 620 | 620 | 310-300 | *W. anomalus* (100%) | A |
| | **Y7,** Y9 | 450 | 350-100 | 310-310 | *C. ethanolica* (83%) | E |
| | **Y47, Y51** | 800 | 800 | 410-380 | *T. delbrueckii* (100%) | C |
| | **Y21, Y40,** Y41, Y42, Y43, Y44, Y48, Y49, Y50, Y52, | 880 | 325-230-170-125 | 375-365-110 | *S. cerevisiae* (100%) | B |
| M4 | Y16, Y20, Y39 | 620 | 620 | 310-300 | *W. anomalus* (100%) | A |
| | **Y15, Y17, Y18, Y19, Y27,** Y28, Y29, **Y30,** Y31, Y32, Y33, Y34, Y35, Y36, Y37, Y38 | 880 | 325-230-170-125 | 375-365-110 | *S. cerevisiae* (100%) | B |
| | **Y6,** Y11 | 630 | 420-130-80 | 310-310 | *C. maritima* (100%)/ *C. zeylanoides*/*C. diddensiae* (100%) | F |

Tentative yeast identification was obtaining by using ITS restriction patterns as queries in Yeast ID databases (www.yeast-id.org). Strains in bold were submitted to sequencing of the 26S rDNA D1/D2 domain. All the fragment sizes are in bp. Fragments lower than 70 bp were omitted from the analysis.

**Table S5.** 26S rRNA D1/D2 region sequencing results and BLAST search on NCBI RefSeq database (28S ribosomal RNA Nucleotide sequence records).

| ITS PCR-RFLP Pattern | Strain | Accession number | BLASTn search | | | |
|---|---|---|---|---|---|---|
| | | | Query cover (%) | *E*-value | Identity (%) | Best Hits |
| A | Y1 | MN058031 | 100 | 0.0 | 100 | *W. anomalus* strain CBS5759 (MK394130.1) |
| | Y67 | MZ221134 | 100 | 0.0 | 100 | *W. anomalus* strain CBS5759 (MH545921.1) |
| | Y56 | MZ221130 | 100 | 0.0 | 100 | *W. anomalus* strain CBS5759 (MH545921.1) |
| | Y59 | MZ221132 | 100 | 0.0 | 100 | *W. anomalus* strain CBS5759 (MH545921.1) |
| | Y60 | MZ221133 | 100 | 0.0 | 100 | *W. anomalus* strain CBS5759 (MH545921.1) |
| B | Y15 | MN058035 | 100 | 0.0 | 100 | *S. cerevisiae* strain CEN.PK113-7D (CP0460921) |
| | Y17 | MN058036 | 100 | 0.0 | 100 | *S. cerevisiae* strain CEN.PK113-7D (CP0460921) |
| | Y18 | MN058037 | 100 | 0.0 | 100 | *S. cerevisiae* strain CEN.PK113-7D (CP0460921) |
| | Y19 | MN058038 | 100 | 0.0 | 100 | *S. cerevisiae* strain CEN.PK113-7D (CP0460921) |
| | Y21 | MN058039 | 100 | 0.0 | 100 | *S. cerevisiae* strain CEN.PK113-7D (CP0460921) |
| | Y23 | MN058040 | 100 | 0.0 | 100 | *S. cerevisiae* strain CEN.PK113-7D (CP0460921) |
| | Y26 | MN058041 | 100 | 0.0 | 100 | *S. cerevisiae* strain CEN.PK113-7D (CP0460921) |
| | Y27 | MZ221124 | 100 | 0.0 | 100 | *S. cerevisiae* strain CEN.PK113-7D (CP0460921) |
| | Y30 | MZ221125 | 100 | 0.0 | 100 | *S. cerevisiae* strain CEN.PK113-7D (CP0460921) |
| | Y40 | MZ221126 | 100 | 0.0 | 100 | *S. cerevisiae* strain CEN.PK113-7D (CP0460921) |
| | Y50 | MZ221128 | 100 | 0.0 | 100 | *S. cerevisiae* strain CEN.PK113-7D (CP0460921) |
| | Y58 | MZ221131 | 100 | 0.0 | 100 | *S. cerevisiae* strain CEN.PK113-7D (CP0460921) |
| C | Y47 | MZ221127 | 99 | 0.0 | 100 | *T. delbrueckii* strain CBS2733 (KY109859.1) |
| | Y51 | MZ221129 | 99 | 0.0 | 100 | *T. delbrueckii* strain CBS2733 (KY109859.1) |
| | Y70 | MZ221135 | 99 | 0.0 | 100 | *T. delbrueckii* strain CBS2733 (KY109859.1) |
| D | Y5 | MN058032 | 100 | 0.0 | 100 | *C. boidinii* strain CBS6202 (KY106345.1) |
| E | Y7 | MN058034 | 100 | 0.0 | 100 | *P. kluyveri* strain CBS7275 (KY108826.1) |
| F | Y6 | MN058033 | 100 | 0.0 | 100 | *C. diddensiae* strain CBS2214 (MK394116.1) |
